# Rheological Performance of High-Temperature-Resistant, Salt-Resistant Fracturing Fluid Gel Based on Organic-Zirconium-Crosslinked HPAM

**DOI:** 10.3390/gels9020151

**Published:** 2023-02-11

**Authors:** Hui Xin, Bo Fang, Luyao Yu, Yongjun Lu, Ke Xu, Kejing Li

**Affiliations:** 1 Shanghai Key Laboratory of Multiphase Materials Chemical Engineering, Lab of Chemical Engineering Rheology, Research Center of Chemical Engineering, East China University of Science and Technology, Shanghai 200237, China; 2 China Petroleum Exploration and Development Research Institute, Beijing 100083, China

**Keywords:** HPAM, fracturing fluid gel, high-temperature-resistant, salt-resistant, rheological analysis, zirconium crosslinker, molecular dynamics simulation

## Abstract

Development of low-cost, high-temperature-resistant and salt-resistant fracturing fluids is a hot and difficult issue in reservoir fluids modification. In this study, an organic zirconium crosslinker that was synthesized and crosslinked with partially hydrolyzed polyacrylamide (HPAM) was employed as a cost-effective polymer thickener to synthesize a high-temperature-resistant and salt-resistant fracturing fluid. The rheological properties of HPAM in tap water solutions and 2 × 10^4^ mg/L salt solutions were analyzed. The results demonstrated that addition of salt reduced viscosity and viscoelasticity of HPAM solutions. Molecular dynamics (MD) simulation results indicated that, due to electrostatic interaction, the carboxylate ions of HPAM formed an ionic bridge with metal cations, curling the conformation, decreasing the radius of rotation and thus decreasing viscosity. However, optimizing fracturing fluids formulation can mitigate the detrimental effects of salt on HPAM. The rheological characteristics of the HPAM fracturing fluid crosslinking process were analyzed and a crosslinking rheological kinetic equation was established under small-amplitude oscillatory shear (SAOS) test. The results of a large-amplitude oscillation shear (LAOS) test indicate that the heating effect on crosslinking is stronger than the shear effect on crosslinking. High-temperature-resistant and shear-resistant experiments demonstrated good performance of fracturing fluids of tap water and salt solution at 200 °C and 180 °C.

## 1. Introduction

Hydraulic fracturing technology is an important means of oil and gas reservoir exploration, evaluation, stimulation and stable production [1,2]. In hydraulic fracturing, chemicals of high viscosity are pumped into the formation under high pressure to generate cracks and obtain new channels for oil recovery by enlarging the fractures to boost oil output in the field. Therefore, the key to fracturing technique is fracturing fluid [3]. Due to the highly uneven distribution of oil and gas resources in China, poor geological conditions and a low ratio of oil and gas resources in conventional reservoirs, low-permeability, deep, marine and unconventional reservoirs have become the focus of oil exploration [4]. Especially as drilling progresses to deep and ultra-deep wells, the temperature rises as the depth of the reservoir grows. Hence, the fracturing process requires a fracturing fluid with high-temperature resistance. Water-based fracturing fluids [5] include synthetic polymer fracturing fluids [2,6,7,8,9], bio-based fracturing fluids [10,11,12] and viscoelastic surfactant (VES) fracturing fluids [13,14,15] as the main fracturing fluids suitable for high temperatures. However, poor thermal stability of glycosidic bonds of biopolymers [6,16] and the high cost of VES fracturing fluids due to high loading have led to limited application at higher temperatures. In 2023, a novel VES fracturing production integral fluid based on cetyltrimethylammonium chloride, salicylic acid and oleic acid amide methyl hydroxypropyl ammonium chloride has been reported by Yan et al. [17] and attracted much more attention. The fluid possesses comprehensive functions of fracturing and oil flooding, with temperature- and salt resistance at 90 °C, outstanding emulsification effect, good oil displacement ability and static oil washing rate. This kind of VES fluid reduces the cost of VES fracturing material resources [17]. Thus far, in the research field of high-temperature resistance, especially under ultra-high-temperature conditions (reservoirs over 80 °C are considered as high-temperature reservoirs and reservoirs above 120 °C are considered as ultra-high-temperature reservoirs [18]), synthetic polymers of greater reconfigurability and lower loading have been generated, making polymer fracturing fluids more promising for high-temperature resistance. On the other hand, water-based fracturing fluids require significant quantities of fresh water, requiring an average of $1.5\times 10^{4}\;$ m^3^ of water per well to meet fracturing requirements [19]. In instances where the water source is not close to the well site, additional water must be transported to prepare the fracturing fluid, making operations more difficult and expensive; in other instances, such as drought-stricken regions, offshore platforms and desert areas, it is even impractical to secure sufficient freshwater resources. To solve this problem, consideration is gradually being given to recycling of produced water from oil fields and use of seawater to tackle the problem of water resources. Thus, it is vital to consider salt resistance performance while ensuring temperature resistance [20,21,22,23]. Given the growing number of reservoirs with harsh natural characteristics (high temperature and high mineralization), use of traditional fracturing fluids faces new challenges and restrictions. How to increase high-temperature performance and salt resistance of fracturing fluids cost-effectively is a significant challenge for both industry and academia.

Acrylamide-based polymers are the most commonly utilized polymers in fracturing fluids, and introduction of functional monomers, such as rigid monomer (4-isopropenylcarbamoyl-benzene sulfonic acid (AMBS) [24], 2-acrylamido-2-methylpropanesul-sulfonic acid (AMPS) [25,26], N-vinyl-2-pyrrolidone (NVP) [26,27]), hydrophobic monomer (N-(3-methacrylamidopropyl)-N,N-dimethyldodecan-1-aminium [8,27], dimethyl diallyl ammonium chloride (DMDAAC) [28]) and thermoviscosifying monomer (such as N-isopropylacrylamide [9]), etc., by radical copolymerization [24,25,26], fragmentation chain transfer polymerization (RAFT) process [7,27] or grafting [9] can substantially improve temperature- and salt resistance capabilities. Another more cost-effective way to achieve temperature- and salt resistance is by adding additives such as crosslinkers and temperature stabilizers to the polymer. Crosslinkers increase elasticity of fracturing fluids without increasing polymer concentration [23]. Some metal crosslinkers (such as zirconium (Zr), titanium (Ti) and aluminum (Al) crosslinkers) can be crosslinked with acrylamide-based polymers to different pH levels, and these metal ions are typically chelated with organic ligands to induce delayed crosslinking [29]. Heat stabilizers (oxygen scavengers) enable scavenging oxygen radicals in base water and protect gel from degradation caused by divalent or trivalent ions or oxidation processes. Methanol and sodium thiosulfate are the two most commonly used heat stabilizers [30]. Many co-polymers crosslinked with Zr crosslinkers have achieved good high-temperature resistance and salt resistance. Zhang et al. [24] synthesized a terpolymer (MAS-1) by free radical copolymerization using acrylamide (AM), acrylic acid (AA) and AMBS as monomers. The viscosity of fracturing fluids containing 0.3 wt% MAS-1 was found to be about 135 mPa·s after 120 min at 150 °C. Holtsclaw et al. [25] developed a new hydraulic-fracturing fluid, which is a ternary polymer (AM, AA and AMPS) crosslinked with metal crosslinker, that is capable of reaching fluid service temperatures up to 232 °C. Yan et al. [28] used AM, AA, sodium p-styrenesulfonate (PS) and DMDAAC as monomers to obtain high-temperature-resistant polymers via free radical copolymerization, and the polymer fracturing fluid maintained good temperature resistance at 180 °C. Xu et al. [26] prepared a new AM/N, N-dimethylacrylamide (DMAM)/AMPS/AA/NVP five-element polymer fracturing fluid system with temperature resistance of 245 °C. Although these copolymers have excellent hydration, high-temperature-, salt- and shear resistance, use of numerous functional monomers complicates polymer synthesis and runs opposed to the initial goal of cost-effectiveness in oilfield applications. Hydrolyzed polyacrylamide (HPAM), as the simplest derivative of acrylamide polymer, has received attention by crosslinking with metal crosslinkers to form temperature-resistant fracturing fluids [31]. From the standpoint of fracturing fluid formulation, Almuarak et al. [32,33] examined the extent that additives, such as oxygen scavengers, crosslinkers, crosslinker delay additives and pH buffers, contribute to rheological characteristics and thermal stability of polymeric fracturing fluids, demonstrating that the main bonds that can influence thermal stability of fracturing fluids are polymer backbone bonds and crosslinker-to-polymer bonds. Furthermore, addition of oxygen scavengers to the fracturing fluid system is also advantageous for temperature resistance performance. This view demonstrates the importance of formulation on thermal stability of fracturing fluids.

In this study, in order to obtain high-temperature resistance and salt resistance fracturing fluids more economically, we employed HPAM with a high molecular weight as a thickener and examined the effect of salt on HPAM solutions using rheological techniques. Molecular dynamics simulation is performed to study molecular behavior and mechanism of HPAM in water solutions and different salt solutions. Further, the crosslinking process of polymer fracturing fluid has also attracted interest of researchers, but there are relatively few studies on it [34,35]. The fracturing fluid formula is optimized, the influence of temperature and strain on the crosslinking process of fracturing fluid is explored under small-amplitude oscillatory shear (SAOS) tests and a primary reaction rheological kinetic equation is established. The rheological phenomenon of non-isothermal crosslinking process is discussed under large-amplitude oscillatory shear (LAOS) tests. Finally, the high-temperature resistance and shear resistance of the fracturing fluid are evaluated. Novel HPAM fracturing fluid with a temperature resistance of 200 °C in tap water and a temperature resistance of 180 °C in 2 × 10^4^ mg/L salt solution is obtained.

## 2. Results and Discussion

### 2.1. Apparent Viscosity of HPAM Solution

**Figure 1:**
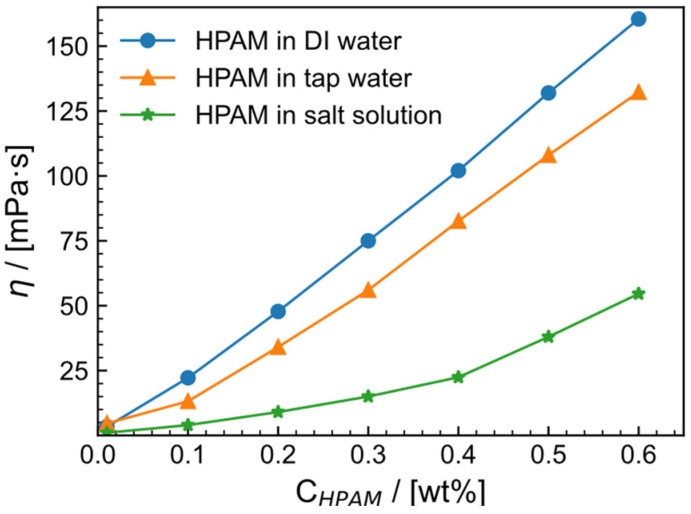
The apparent shear viscosity (η) of HPAM in deionized (DI) water, tap water and 2 × 10^4^ mg/L (1.5 × 10^4^ mg/L NaCl and 0.5 × 10^4^ mg/L CaCl_2_) salt solution at 30 °C and shear rate of 100 s^−1^.

Apparent viscosity reflects the ability of the fracturing fluid to carry the proppant. However, the viscosity of the HPAM solution is affected by factors such as shear rate, salinity and concentration. In order to meet the actual construction needs of the fracturing fluid, different mass fractions (0.01–0.6 wt%) of HPAM solutions were prepared in deionized water, tap water and in 2 × 10^4^ mg/L (1.5 × 10^4^ mg/L NaCl and 0.5 × 10^4^ mg/L CaCl_2_) salt solution. The viscosity values of the solutions and their corresponding concentrations are shown in Figure 1. In the test range, the viscosity of the HPAM solution increases with increasing concentration in these different solutions [36]. Due to the presence of trace minerals in tap water and the influence of Na^+^ and Ca^2+^ in salt solution, the viscosity of the HPAM solution in tap water and salt solution with the same concentration was significantly lower than the viscosity of the HPAM solution in deionized water. Therefore, the corresponding relationship between the viscosity of the same concentration is deionized water > tap water > salt solution. To ensure good efficacy of the fracturing fluid, HPAM solution with a concentration of 0.6 wt% was used as the thickener. In tap water solution, the apparent viscosity of HPAM is 132.28 mPa·s, while, in salt solution, it is 54.59 mPa·s.

### 2.2. Flow Curve of HPAM Solution

**Figure 2:**
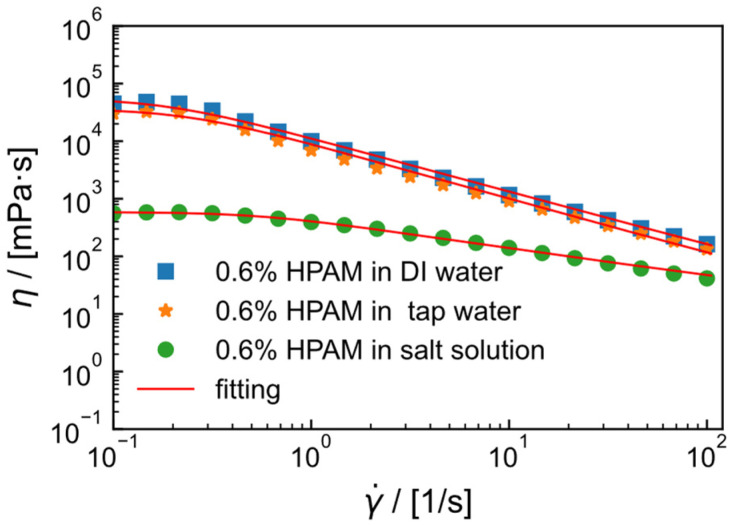
Viscosity (η) as a function of shear rate ($\dot{\gamma }$ ) of 0.6 wt% HPAM in deionized (DI) water, tap water and 2 × 10^4^ mg/L (1.5 × 10^4^ mg/L NaCl and 0.5 × 10^4^ mg/L CaCl_2_) salt solution at 30 °C. Solid lines represent the best fit for the Carreau model.

In different salt solutions, the apparent viscosity of 0.6 wt% HPAM at different shear rates was measured, resulting in the flow curves shown in Figure 2. At low shear rates, the viscosity of HPAM solution remains constant, while, at high shear rates, the fluid exhibits power-law behavior. All flow curves are fitted with the Carreau model (Equation (1)), which is suitable for shear-thinning fluids and can be used to describe polymer solutions.

(1) $$\eta -\eta_{\infty }=\left(\eta_{0}-\eta_{\infty }\right){\left[1+{\left(\lambda \dot{\gamma }\right)}^{2}\right]}^{\frac{n-1}{2}},$$

where $\eta_{\infty }$ is infinite shear viscosity, $\eta_{0}$ is zero shear viscosity, n is the power law slope and λ is the characteristic time, the inverse of which represents the shear rate at which the transition from Newtonian behavior to shear thinning occurs.

The fitting parameters of the Carreau model are shown in Table 1, with correlation coefficients all close to 0.99, indicating good fitting performance. In the model, the characteristic time λ can represent the dispersion and flowability of polymer solutions. The longer the characteristic time, the better the dispersion and flowability [37]. The characteristic time of HPAM in deionized water solution is longer (4.73 s) compared to that in tap water (4.29 s), while it is shortest in salt solution (1.82 s), indicating that the dispersion of HPAM is better in deionized water and worse in salt solution. In addition, some studies have also used the Carreau–Yasuda model to fit and summarize the flow curves of dilute HPAM solutions [38].

### 2.3. Viscoelasticity of HPAM Solution

**Figure 3:**
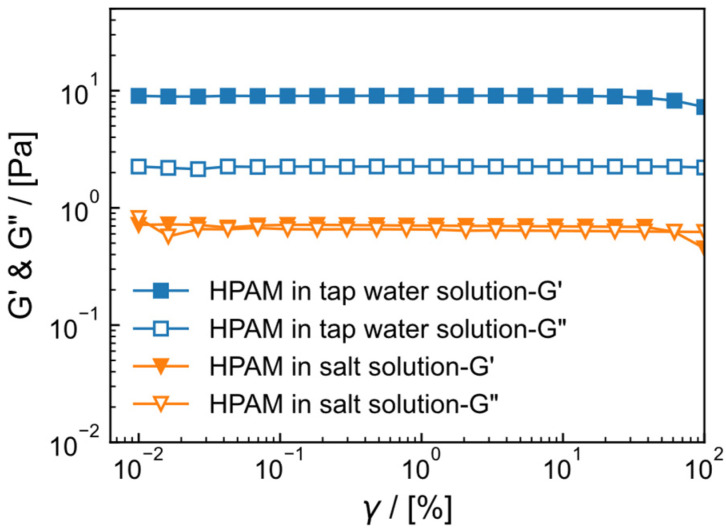
The storage modulus ($G{}^{\prime }$) and loss modulus ($G{}^{\prime \prime }$) of 0.6 wt% HPAM at different strains (γ) in tap water and 2 × 10^4^ mg/L (1.5 × 10^4^ mg/L NaCl and 0.5 × 10^4^ mg/L CaCl_2_) salt solution at 30 °C and *f* = 1 Hz.

The storage modulus ($G{}^{\prime }$) and loss modulus ($G{}^{\prime \prime }$) of 0.6 wt% HPAM in tap water and salt solution are shown in Figure 3. When the shear strain ( γ) ranges from 0.01% to 100% with a frequency f = 1 Hz, the boundary between the platform region and the downward region of the strain scan is used to divide the linear zone and the nonlinear zone. Both HPAM in tap water and salt solution have a relatively long linear viscoelastic region, which is γ = 0.01–50%. At 0.6 wt% HPAM in tap water, $G{}^{\prime }\;$ is maintained at 9.04 Pa, $G{}^{\prime \prime }$ is maintained at 2.25 Pa, $G{}^{\prime }>G{}^{\prime \prime }$ and there is no intersection between the two within the test range, indicating that the system elasticity dominates and the solution structure is stronger. For the salt solution system, $G{}^{\prime }$ and $G{}^{\prime \prime }$ are significantly lower than those in the tap water system, with $G{}^{\prime }$ maintained at 0.71 Pa and $G{}^{\prime \prime }$ maintained at 0.65 Pa. At a strain of 61.4%, $G{}^{\prime }=G{}^{\prime \prime }$, indicating that the structure of HPAM in the salt solution system is weaker. Viscoelasticity reflects the density of the polymer network in solution to some extent [24]. The decrease in viscoelasticity of HPAM in salt solution suggests that the network density of HPAM in salt solution is less than that of HPAM in tap water solution, demonstrating that salt has a negative impact of salt on the structure of HPAM.

### 2.4. Conformation and Behavior of HPAM Solution

**Figure 4:**
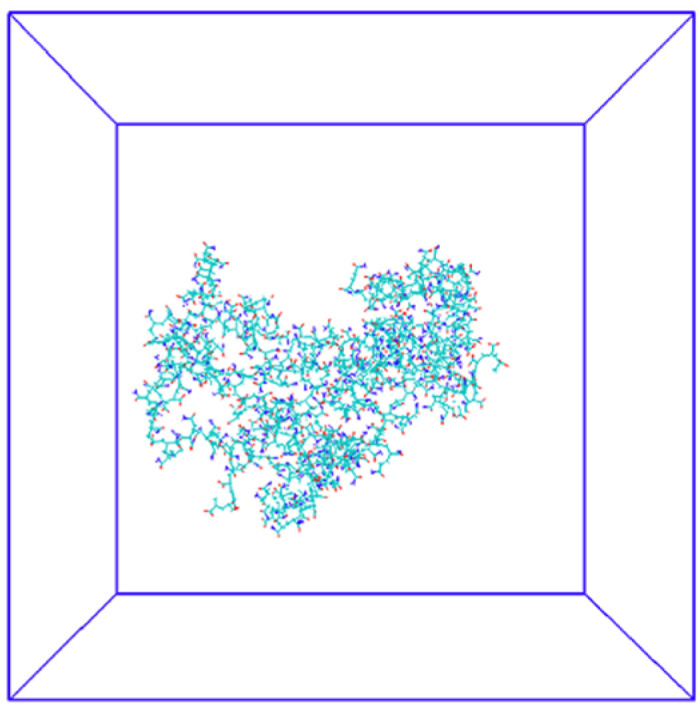
HPAM initial conformation. Cyan represents carbon atoms, red represents oxygen atoms and blue represents nitrogen atoms.

**Figure 5:**
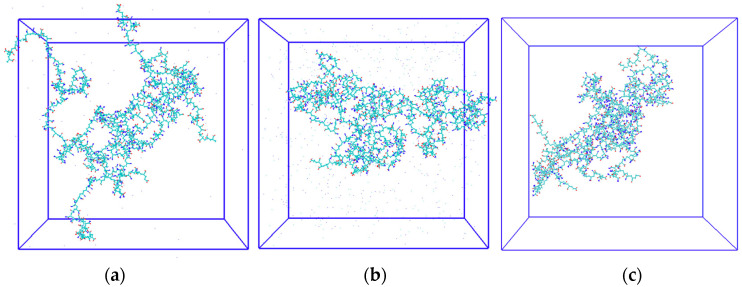
The conformation of HPAM after 50 ns simulation in (**a**) water solution, (**b**) NaCl solution and (**c**) CaCl_2_ solution at 300 K and 1 atm.Cyan represents carbon atoms, red represents oxygen atoms and blue represents nitrogen atoms.

To further understand the mechanism of HPAM behavior in different solutions, the initial structure of HPAM is shown in Figure 4, and the molecular structure of HPAM after simulating 50 ns in different solvent environments with the same initial structure is shown in Figure 5. Figure 5a shows HPAM in water (with a very low concentration of added Na^+^ to make the system neutral), Figure 5b shows HPAM in a NaCl salt solution (0.342 mol/L) and Figure 5c shows HPAM in a CaCl_2_ solution (0.342 mol/L). As shown in Figure 5a, molecular chain of HPAM expands in water due to the electrostatic repulsion between charged monomers (-COO^−^) along the main chain of HPAM, which maximizes the diffusion volume. In contrast, as shown in Figure 5b, c, HPAM exhibits different degrees of curling in the two salt solutions and the curling is stronger in the CaCl_2_ solution than in the NaCl solution. This is due to the strong electrostatic attraction between the charged carboxyl group and the cations, which attracts the cations to the polymer chain segments and compresses the polymer molecular chain segments, resulting in shrinkage of the polymer chain, and the effect of divalent cations is stronger than that of monovalent cations.

### 2.5. Gyration Radius of HPAM Solution

Radius of gyration (Rg) is a characteristic parameter of linear polymers that specifies the spatial extension of the polymer chain and directly reflects the chain’s conformation. By determining the mean square distance between the center of mass and the center of mass of the atoms, Rg can be utilized to quantitatively quantify the effect of salt on the structure of HPAM. Rg is defined by Equation (2).

(2) $$Rg=\sqrt{\frac{\sum_{i=1}^{n}m_{i}R_{i}^{2}}{\sum_{i=1}^{n}m_{i}}},$$

where $m_{i}$ is the mass of each chain unit and $R_{i}$ is the distance between the polymer chain’s mass center and the ith (i=1,2,3,…,n) chain unit.

**Figure 6:**
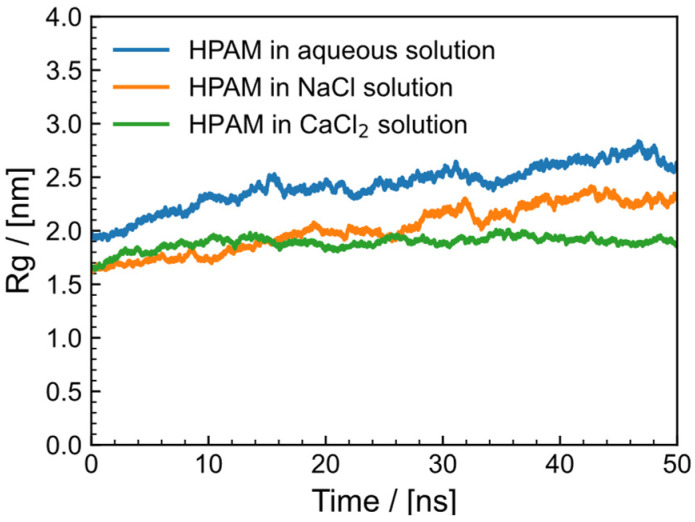
Radius of rotation of HPAM in different solutions (aqueous, NaCl solution and CaCl_2_ solution).

As shown in Figure 6, the average Rg was utilized to quantify the structural changes of each HPAM polymer chain in different solvent conditions (water, NaCl solution and CaCl_2_ solution) during the simulation procedure. The average Rg of HPAM in water was approximately 2.5 nm, in NaCl solution it was approximately 2 nm and in CaCl_2_ solution it was approximately 1.7 nm. This indicates that HPAM is not easily extended in salt solutions, and CaCl_2_ has a larger impact on HPAM than NaCl. Chen et al. [39] simulated the Rg of single PAM and HPAM chains in different concentrations of NaCl and discovered that Rg decreases as salt concentration increases. Du et al. [40] compared the Rg of HPAM, AM/AMPS and AM-NVP in mixed salt solutions of Na^+,^ Mg^2+^ and Ca^2+^ but did not compare the Rg of HPAM in different types of salt solutions. In addition, it is difficult to determine whether the system has reached equilibrium using conventional methods for flexible molecules. The structure of the polymer in aqueous solution will constantly change, but the overall change is minimal, indicating that the system has reached equilibrium.

### 2.6. Ionic Bridge

**Figure 7:**
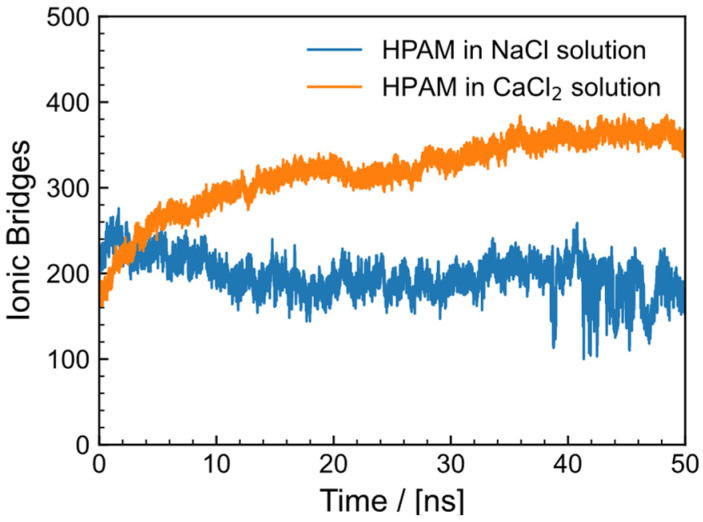
Number of salt bridges between O atom in -COO^−^ group of HPAM and cation (Na^+^ and Ca^2+^).

To further investigate the interaction between HPAM and metal ions, the number of salt bridges between -COO^−^ and Na^+^ and Ca^2+^ at the same concentration was determined. A strong electrostatic contact between positive and negative charges that binds two atomic groups constitutes an ionic bridge. Typically, the distance between counterions is smaller than 4.5 Å, indicating formation of an ion bridge. The amount of ion bridges formed by HPAM in NaCl and CaCl_2_ solutions fluctuates with simulation time, as depicted in Figure 7. The average number of salt bridges formed by the O atom in -COO^−^ group of HPAM with Ca^2+^ remained at 318, but the stability of salt bridges formed with Na^+^ was 197. At same ions concentration, the carboxyl group produced nearly 1.6 times as many ionic bridges with Ca^2+^ as with Na^+^, indicating that divalent metal ions exert a stronger influence on the carboxyl group. Both Na^+^ and Ca^2+^ can form salt bridges with carboxyl groups. This further demonstrates that addition of metal cations has an electrostatic shielding effect on the -COO^−^ group, resulting in a decrease in the Rg of HPAM. Simultaneously, the salt generates an ion layer to reduce the interaction between HPAM and water, thereby thinning the hydration layer around HPAM and decreasing the dispersibility of the polymer chain, resulting in a drop in viscosity [41,42].

### 2.7. Effect of Salt Solution on HPAM Fracturing Fluid

Although salt has a negative effect on the hydration properties of HPAM solutions, the effects of other additives, such as pH control agent, crosslinkers and heat stabilizers, must be considered when formulating polymeric fracturing fluids. In this section, we utilized 0.5 mol/L HCl as the pH control agent, zirconium chelated with triisopropylamine and lactic acid as the organic Zr crosslinker and methanol as the temperature stabilizer to synthesize HPAM fracturing fluids and evaluated the differences in the properties of aqueous and salt solution HPAM fracturing fluids.

Dynamic rheology is a valuable tool for monitoring crosslinking and microstructure, and measuring within the linear viscoelastic area under SAOS tests can detect changes in the gel’s internal structure without damaging it. To explore the influence of pH crosslinker and crosslinker on HPAM fracturing fluid and to establish optimal formulation conditions, the modulus at  f=1 Hz and γ = 10% (the linear viscoelastic region γ = 0.1–50%) was chosen as the evaluation index for gel strength.

#### 2.7.1. Effect of pH Control Agent

**Figure 8:**
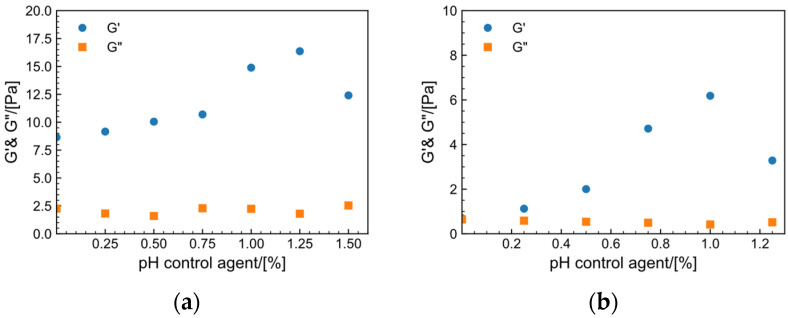
Effect of pH control agent dosage on the storage modulus ($G{}^{\prime }$) and loss modulus ($G{}^{\prime \prime }$) of the system (C_(Zr crosslinker)_ = 1.5%) at 30 °C, f=1 Hz and γ = 10%. (**a**) Tap water system. (**b**) Salt solution system.

The pH control agent is crucial for regulating the release rate of Zr^4+^. When the fixed Zr crosslinker concentration is 1.5 wt% and MeOH is 1 wt%, the effect of the pH control agent on the thickening in the tap water system (a) and the saltwater system (b) is illustrated in Figure 8. When no pH control agent is added, the modulus following addition of the crosslinking agent is comparable to the modulus of the HPAM solution at γ = 10%, as shown in Figure 3. HPAM solution has a pH of 8.07, whereas Zr crosslinkers typically only crosslink under acidic circumstances [43]. Overall, the tap water system has a higher modulus than the salt solution system. $G{}^{\prime }\;$ of the two systems increases and subsequently decreases when the pH control agent is increased, whereas $G{}^{\prime \prime }$ remains almost unchanged, demonstrating that altering the pH range can control release of zirconium ions and that there is an appropriate dose range. The best dosage of pH control agent in tap water is 1.25%, with a modulus of 16.3 Pa, but the optimal dosage in salt water is 1%, with a modulus of 6.2 Pa.

#### 2.7.2. Effect of Crosslinker

**Figure 9:**
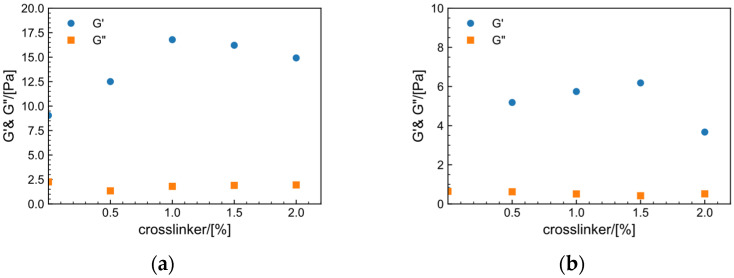
Effect of Zr crosslinker dosage on the storage modulus ($G{}^{\prime }$) and loss modulus ($G{}^{\prime \prime }$) of the fracturing fluid gel (C_(pH control agent)_ = 1.25%) at 30 °C, f=1 Hz and γ = 10%. (**a**) Tap water system. (**b**) Salt solution system.

After determining the amount of pH control agent, further explore the effect of the amount of crosslinker on the gel performance. As illustrated in Figure 9, the effect of the crosslinker on the thickening agent of the tap water system (a) and the saltwater system (b) is investigated when the concentration of pH control agent is held constant at 1.25 wt% and MeOH at 1 wt%. The optimal amount of Zr crosslinker in the tap water system is 1.0% and the $G{}^{\prime }$ is 16.7 Pa, whereas the best amount in the saltwater system is 1.5 wt% and $G{}^{\prime }$ is 6.2 Pa. When the amount of Zr crosslinker is inadequate, the concentration of zirconium ions per unit volume is low and crosslinking is insufficient. When the crosslinking ratio is excessively high, excessive crosslinking will occur, resulting in a gel structure that breaks easily. After optimization, the formulation of the fracturing fluid gel for the tap water system is 0.6 wt% HPAM + 1.25 wt% 0.5 mol/L HCl + 1 wt% MeOH + 1.0 wt% Zr crosslinker. The formulation of the salt solution system is 0.6 wt% HPAM + 1.0 wt% 0.5 mol/L HCl + 1 wt% MeOH + 1.5 wt% Zr crosslinker.

### 2.8. Microscopic Morphology and Picking Properties of Fracturing Fluid Gels

**Figure 10:**
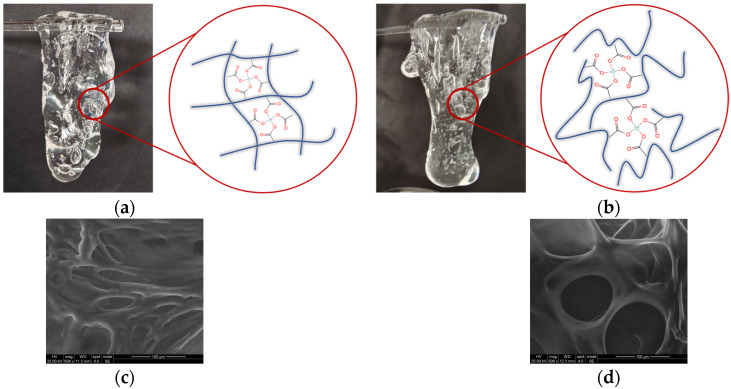
(**a**,**b**) Digital photographs and schematic diagram and (**c**,**d**) microstructure images of the crosslinked HPAM fracturing fluid gel in tap water system and salt solution system.

Using the optimized system formulation according to Section 2.5, additives were added to the polymer solution and agitated with a glass rod for approximately 5 min until they could be picked up. The picking properties performance and schematic diagram of crosslinked structure of fracturing fluid gel obtained by HPAM are shown in Figure 10a for tap water system and Figure 10b or salt solution system. Despite the salt-induced curling of the HPAM polymer molecular chains, the carboxyl groups in HPAM are still capable of crosslinking with Zr crosslinker to form a gel. The gels of both systems have good picking performance. In addition, the macroscopic properties of a gel system are determined by its microstructure. We observed the microstructure of HPAM fracturing fluid gel using cryo-scanning electron microscopy (Cryo-SEM). The microscopic morphology of the gels in the tap water and salt solution systems is shown in Figure 10c,d, respectively. Gel systems with more homogenous and compact three-dimensional network architectures are more stable [35]. All the gels in this system can form a mesh structure, indicating that the HPAM fracturing fluid gel has some salt resistance. However, compared to the gel in the salt solution system, the gels in the tap water system have smaller pores, indicating a more dense and stronger structure.

### 2.9. SAOS

#### 2.9.1. Effect of Strain on Crosslinking Process

Crosslinking is the process of converting a linear polymer from a solution to a gel, which involves combination of carboxyl groups in the polymer with zirconium ions to form a network structure [44]. It is assumed that the increase in the $G^{{}^{\prime }}$ of the system is proportional to the decrease in zirconium ions. When all the released zirconium ions are bound to the carboxyl groups in HPAM, the reaction is considered to be complete. It is assumed that the crosslinking process follows first-order kinetics model [34,45].

(3) $$-\frac{dG^{{}^{\prime }}\left(t\right)}{dt}=kG^{{}^{\prime }}\left(t\right),$$

The oscillating rheological equation of crosslinking:

(4) $$\frac{G^{{}^{\prime }}\left(t\right)-G_{c}^{{}^{\prime }}}{G{{}^{\prime }}_{max}-G_{c}^{{}^{\prime }}}=\left[1-\exp\left(-kt\right)\right],$$

where $G^{{}^{\prime }}\left(t\right)$ is storage modulus of gel, $G{{}^{\prime }}_{c}$ is initial storage modulus, $G{{}^{\prime }}_{max}$ is storage modulus at the equilibrium of the crosslinked gel and k is the rate constant of the crosslinking reaction. Equation (4) can accurately describe the dynamic rheological properties of the polymer system during the crosslinking process.

**Figure 11:**
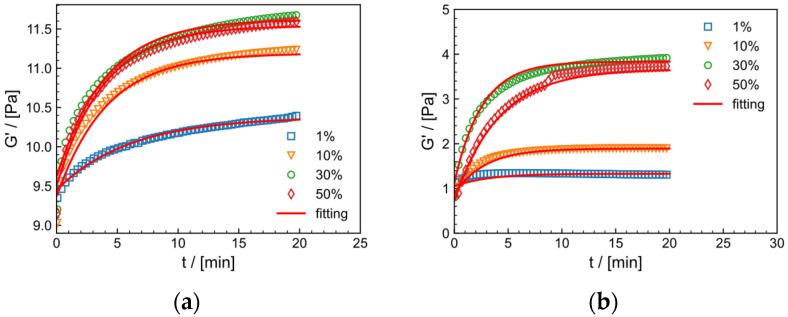
The relationship between the dynamic storage modulus ($G{}^{\prime }$) and time for various strains (1%, 10%, 30%, 50%) in (**a**) the tap water and (**b**) salt solution systems at 30 °C and f=1 Hz.

**Figure 12:**
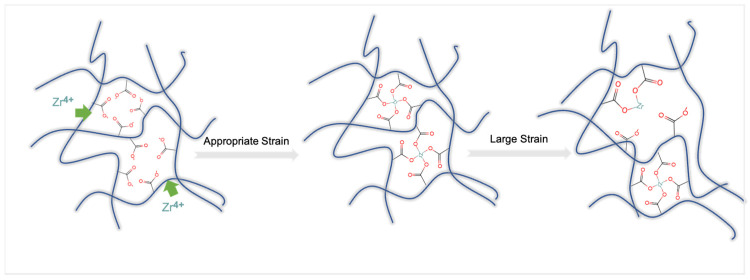
Schematic diagram of effect of shear strain on the interaction of HPAM with organic zirconium crosslinker. Step1, zirconium ions (Zr^4+^) diffuse into HPAM polymer (The green arrow represents the diffusion trend of Zr^4+^). Step2, Zr^4+^ form ligand bonds with carboxyl groups (-COO^−^) of HPAM under appropriate strain. Step3, the cross-linked structure was disrupted under large strain.

The rheological properties of HPAM in tap water system and salt solution system under constant shear frequency (f = 1 Hz) and temperature (30 °C) with strain ranges of 1%, 10%, 30%, 50% are shown in Figure 11a,b, respectively. As the crosslinking process progresses, elastic modulus $G{}^{\prime }$ increases with time at all strains and reaches a steady state after about 10 min, which reflects the delayed crosslinking effect of the crosslinker. Table 2 shows the parameters of Equation (4). Within the strain range of 1–30%,$\;G{}^{\prime }\;$ increases with increasing strain. The k values and $G_{max}^{{}^{\prime }}$ increase and then decrease with increasing strain. This suggests that larger shear strains (within the linear viscoelastic region) help to form the crosslinking network, possibly because larger strains increase the probability of collision between zirconium ions and crosslinking groups, making it easier to form a network. At a strain of 50%, which is close to the nonlinear viscoelastic region, the maximum modulus of both systems decreases, indicating that excessive strain can cause certain damage to the structure, and the gel formation rate is negatively affected. Figure 12 illustrated the stepwise mechanism of the effect of shear strain on the crosslinking process of HPAM. The appropriate strain (γ<50%) has a facilitative influence on the crosslinking process, whereas the gel structure is disrupted beyond the linear viscoelastic zone (γ≥50%).

#### 2.9.2. Effect of Temperature on Crosslinking Process

**Figure 13:**
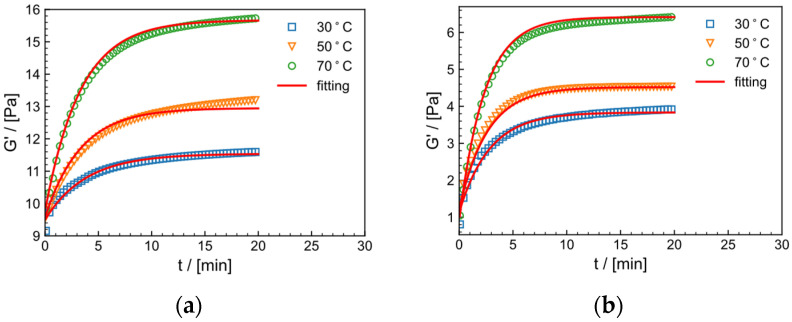
The relationship between dynamic storage modulus ($G{}^{\prime }$) and time for various temperatures (30 °C, 50 °C, 70 °C) at 30 °C and f=1 Hz. in (**a**) the tap water system and (**b**) salt solution systems.

As shown in Figure 13, the crosslinking process of HPAM (1) in tap water and (2) salt solution at the same strain (γ = 30%) and different temperatures (30 °C, 50 °C, 70 °C) is shown. The storage modulus increases significantly with time at each temperature. The fitting parameters of the curve is shown in Table 3. As temperature increases, the structural rate constant k and $G_{max}^{{}^{\prime }}$ of the system increase, which may be due to the increase in collision of crosslinking points at higher temperature, indicating that heating is beneficial for crosslinking. Notably, the k values for the tap water system are all lower than those for the salt solution system.

### 2.10. LAOS

Based on the influence of strain and temperature on the crosslinking process under SAOS tests, it can be noted that an increase in strain (beyond the linear viscoelastic region) disrupts the crosslinking process, whilst an increase in temperature facilitates it. LAOS was utilized to characterize the crosslinking process in order to investigate the rivalry between shear and temperature on the crosslinking process. Using this method as opposed to measuring viscosity (shear mode) has the advantage of ensuring that the fluid is in the nonlinear viscoelastic region as the fracturing fluid frequently undergoes large deformation during injection into the wellbore while preventing measurement errors caused by wall slip and Weissenberg effects during the measurement process. $G^{{}^{\prime }}$ and $G{}^{\prime \prime }\;$ are strictly defined only within the linear viscoelastic range in LAOS. As a result, their values at large strain amplitudes may have vague physical meaning. However, measuring $G^{{}^{\prime }}\left(\gamma \right)$ and $G{}^{\prime \prime }\left(\gamma \right)$ (no longer as a constant but as a function of strain (γ) at a fixed frequency can provide meaningful information [46].

Lissajous curves are plots of stress σ(t) versus strain γ(t), which enable more intuitive study of the waveform characteristics of σ(t) during the crosslinking process. Sinusoidal shear strain and shear stress can be used to obtain the equation to describe the Lissajous curve [46]:

(5) $$\{\begin{array}{c} \gamma \left(t\right)=\gamma_{0}sin\omega t\\ \sigma \left(t\right)=\underset{n}{\sum }\sigma_{n}sin\left(n\omega t+\delta_{n}\right)\; \end{array},\;$$

where t is time, $\gamma_{0}$ is shear strain amplitude, ω is angular frequency, $\sigma_{n}$ is the n-th harmonic amplitude value of shear stress (n=1,odd), $\delta_{n}$ is the n-th harmonic phase angle of shear stress (n=1,odd).

**Figure 14:**
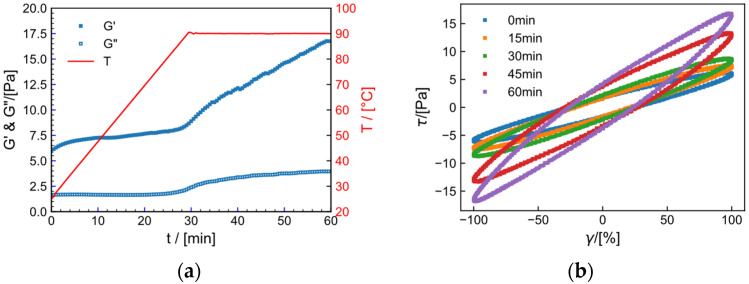
(**a**) Nonlinear storage modulus $G_{1}^{{}^{\prime }}$ and loss modulus $G_{1}{}^{\prime \prime }$ and (**b**) Lissajous curve with time (t) and temperature (T) of hydraulic fracturing fluid system in tap water at γ = 100%.

**Figure 15:**
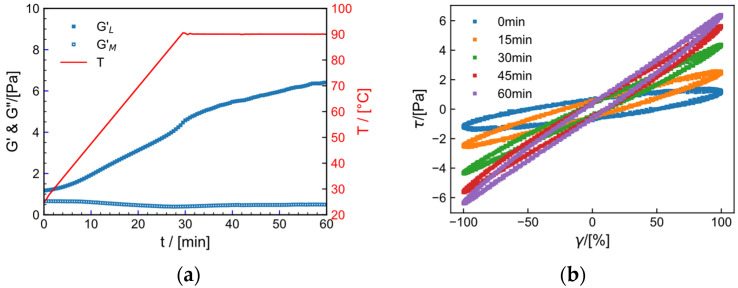
(**a**) Nonlinear storage modulus $G_{1}^{{}^{\prime }}$ and loss modulus $G_{1}{}^{\prime \prime }$ and (**b**) Lissajous curve with time (t) and temperature (T) of hydraulic fracturing fluid system in salt solution at γ = 100%.

Figure 14 and Figure 15 show the time- and temperature-dependent curves of fundamental frequency nonlinear storage modulus $G_{1}^{{}^{\prime }}$ and loss modulus $G_{1}{}^{\prime \prime }$ and Lissajous curve taken at 30 min intervals under strain  γ= 100% for the fracturing fluid in the tap water system and the salt solution system, respectively. In the non-isothermal crosslinking process, $G_{1}^{{}^{\prime }}$ is always greater than $G_{1}{}^{\prime \prime }$ in both systems, as shown in Figure 14a and Figure 15a. The increase in $G_{1}^{{}^{\prime }}$ can be divided into two stages. When the temperature rises from 25 °C to 75 °C, $G_{1}^{{}^{\prime }}$ increases slowly, indicating that the effect of shear on breaking crosslinking and the effect of temperature increase on promoting crosslinking are equivalent. When the temperature rises from 75 °C to 90 °C, $G_{1}^{{}^{\prime }}$ increases significantly, which proves that the promotion effect of temperature on crosslinking is more obvious in this stage. A Lissajous curve is a simplified way to characterize material properties in LAOS testing. Since $G_{1}^{{}^{\prime }}$ and $G_{1}{}^{\prime \prime }$ are not enough to fully represent nonlinear viscoelasticity, the shape of a Lissajous curve can provide additional information. During the crosslinking process, the Lissajous curve shows a perfect elliptical shape, indicating that the system is mainly elastic during the crosslinking process. As time increases, the stress of the Lissajous curve increases accordingly, indicating that elasticity increases gradually. The non-isothermal large-amplitude oscillatory shear test shows that, even under higher strain, the gel can still be crosslinked and its strength increases gradually, which, to some extent, indicates that higher temperature and appropriate large shear are conducive to crosslinking of the gel.

In order to quantitatively describe the characteristics of the Lissajous curve, Ewoldt et al. [47] proposed maximum strain modulus $G_{L}^{{}^{\prime }}$ and minimum strain modulus $G_{M}^{{}^{\prime }}$ to describe the behavior of stress hardening.

(6) $$G_{L}^{{}^{\prime }}={\frac{d\sigma }{d\gamma }|}_{r=\pm \gamma_{0}},\;G_{M}^{{}^{\prime }}={\frac{d\sigma }{d\gamma }|}_{r=0}$$

**Figure 16:**
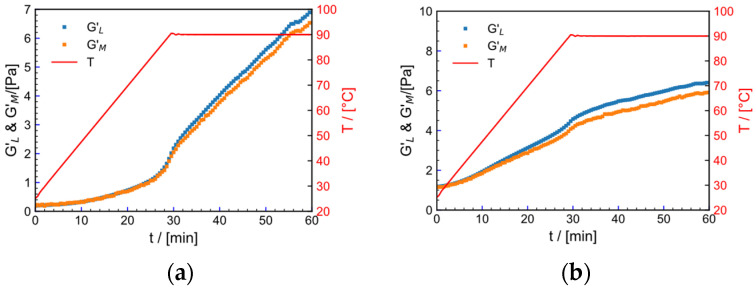
The maximum strain modulus ($G_{L}^{{}^{\prime }}$) and the minimum strain modulus ($G_{M}^{{}^{\prime }}$) as a function of temperature (T) and time (t). (**a**) Tap water system. (**b**) Salt solution system.

Minimum strain modulus $G_{L}^{{}^{\prime }}$ is the modulus at the maximum strain at γ = $\gamma_{0}\;$ and $G_{M}^{{}^{\prime }}$ is the modulus at γ=0. $G_{L}^{{}^{\prime }}$ and $G_{M}^{{}^{\prime }}$ can characterize the shape characteristics of the Lissajous curve. Figure 16 shows the curves of Lissajous curve parameters $G_{L}^{{}^{\prime }}$ and $G_{M}^{{}^{\prime }}$ with temperature and time in the (a) tap water system and (b) salt solution system for the fracturing fluid. The curve can be divided into two stages. In the first stage, for the fracturing fluid in the tap water system, t<24 min, T<50.48 °C, $G_{L}^{{}^{\prime }}\approx G_{M}^{{}^{\prime }}$; for the salt solution system, t<6 min, T<37.6 °C, $G_{L}^{{}^{\prime }}\approx G_{M}^{{}^{\prime }}$, while, in the second stage, $G_{L}^{{}^{\prime }}>G_{M}^{{}^{\prime }}$. This reflects that, in the early stage of heating, temperature and strain have little effect on deformation of the Lissajous curve, but, as temperature increases, the Lissajous curve undergoes slight deformation.

### 2.11. High-Temperature Resistance and Shear Resistance

**Figure 17:**
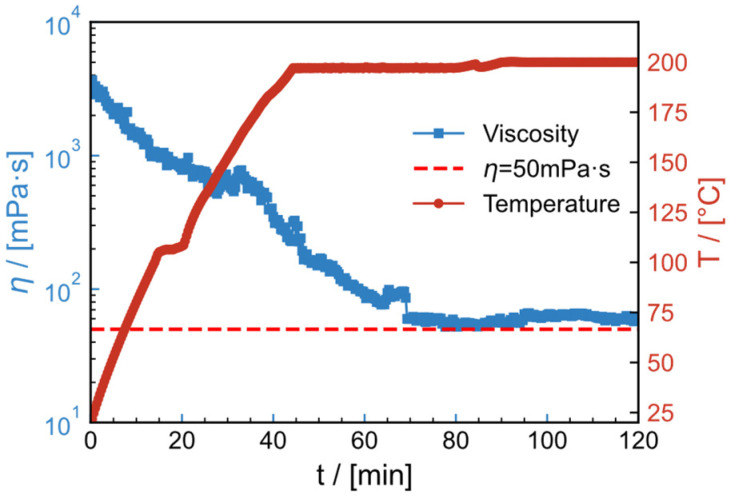
High-temperature resistance and shear resistance curves of 0.6 wt% HPAM polymer fracturing fluid at 200 °C and 100 s^−1^.

**Figure 18:**
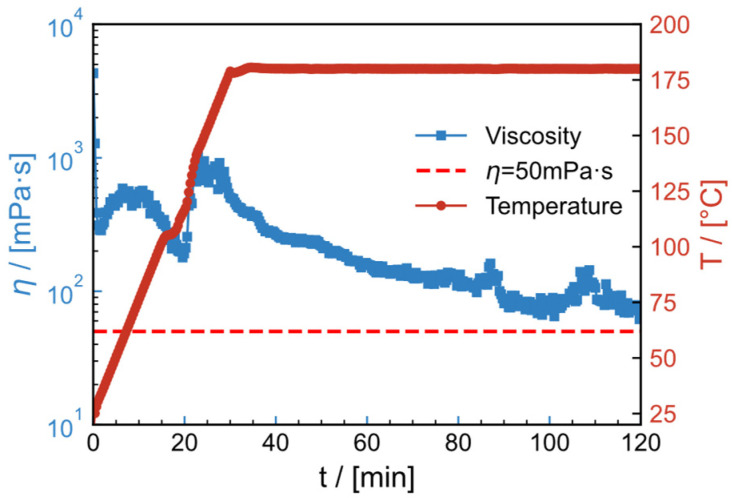
High-temperature resistance and shear resistance curves of 0.6 wt% HPAM polymer fracturing fluid with a salinity of 2 × 10^4^ mg/L (1.5 × 10^4^ mg/L NaCl and 0.5 × 10^4^ mg/L CaCl_2_) at 180 °C and 100 s^−1^.

Figure 17 and Figure 18 illustrate the thermal- and shear resistance curves of the water and salt solution systems, respectively, in order to model the effect of high temperature and shear on fracturing fluids during their injection into the subsurface. The shear rate is 100 s^−1^, the temperature is raised from 25 °C to the specified temperature (200 °C for the water system and 180 °C for the salt solution system) in 30 min and the shear rate is kept constant for 90 min. Viscosity in the water system reduces as temperature rises, eventually stabilizing at 61.43 mPa·s. In the temperature- and shear resistance test at 180 °C, the thermal thickening phenomenon occurs for the salt solution system at 50–70 °C and 100–130 °C during the temperature increase. This may be due to unfolding of the thickener conformation, exposing the carboxyl and causing it to react with the Zr crosslinker. The final viscosity retained is 77.2 mPa·s. All the aforementioned tests satisfy the on-site construction criteria of viscosity greater than 50 mPa·s.

## 3. Conclusions

In this work, rheological techniques are used to examine the influence of salt on HPAM solution and HPAM polymer fracturing fluid. Rheological studies demonstrated that salt solution significantly reduced the viscosity and viscoelasticity of the HPAM solution. Further utilizing MD, the conformation, radius of gyration and interaction mechanism of Na^+^ and Ca^2+^ on HPAM in solution were investigated. The simulation findings revealed that the electrostatic connection between the carboxyl groups in HPAM caused the conformation of HPAM to curl up and the Rg to decrease and that the electrostatic interaction between HPAM and Ca^2+^ was more powerful. The negative effect of salt on HPAM hydration was demonstrated.

To acquire better fracturing fluid formulation, impacts of crosslinker and pH control agent on HPAM in a tap water solution and 2 × 10^4^ mg/L salt solution fracturing fluid system were separately studied. The crosslinking process of fracturing fluids was further discussed systematically under SAOS and LAOS tests, respectively. The results show that crosslinking of HPAM fracturing fluid is facilitated at appropriate temperature (70–90 °C) and shear strain (γ < 50%).

Despite the negative effect of salt on the HPAM solution, the HPAM fracturing fluid gel still has good temperature- and shear resistance after formulation optimization. Tap water system fracturing fluid can reach 200 °C at 100 s^−1^ with a retention viscosity of 61.43 mPa·s during temperature- and shear resistance testing. The temperature- and shear resistance tests for the salt solution system were conducted at 180 °C, and the ultimate retention viscosity was 77.2 mPa·s. This demonstrates that HPAM fracturing fluid is a novel system of high-temperature-resistant and salt-resistant fracturing fluid with outstanding performance under optimal formulation circumstances.

Formulation optimization has a significant impact on temperature resistance of HPAM fracturing fluids. We propose to expand a wider consideration range of fracturing fluid formulations in future work, including HPAM with different hydrolysis degrees and molecular weights. Compatibility of crosslinkers with polymer thickeners can also be discussed in depth.

## 4. Materials and Methods

### 4.1. Materials

Partially hydrolyzed polyacrylamide (HPAM, average molecular weight about 24 million, hydrolysis degree 20–25%) was provided by Beijing Hengju Co., Ltd, Beijing, China. Zirconyl chloride octahydrate (≥98.0%, ZrOCl_2_·8H_2_O), Hydrochloric acid (37.0%, HCl) and methanol (AR, CH_3_OH) were purchased from Sinopharm Chemical Reagent Co., Ltd, Shanghai, China. Triisopropanolamine (99.0%, C_9_H_21_NO_3_) was purchased from Shanghai Boer Chemical Reagent Co., Ltd, Shanghai, China. Lactic acid (AR, C_3_H_6_O_3_) was purchased from Shanghai Lingfeng Chemical Reagent Co., Ltd., Shanghai, China. Tap water was supplied by Shanghai. All chemicals were used directly without further purification.

### 4.2. Preparation of Organic Zirconium Crosslinker

Organic zirconium crosslinker is a metal complex formed by using zirconium as the central ion and triisopropanolamine and lactic acid as ligands. The preparation process was as follows. Six grams of ZrOCl_2_·8H_2_O was added to 54 g of deionized water in a 250 mL flask and heated to 55 °C in a constant temperature water bath at 300 rad/min to allow sufficient hydrolysis. After 3 h of reaction, 3 g of lactic acid and 30 g of triisopropanolamine were added within 10 min, respectively, and the reaction continued for an additional 8 h. After cooling to room temperature, the final product was obtained.

### 4.3. Preparation of Polymer Fracture Fluids Gel

According to the fracturing fluid salt resistance standard (SY/T7627-2021), a salt solution with a mineralization of 2 × 10^4^ mg/L is prepared (NaCl and CaCl_2_, where CaCl_2_ is 500 mg/L). The polymer (HPAM) is completely dissolved in the salt solution or tap water to obtain a polymer solution with a mass fraction of 0.0–0.6% as a thickener. To generate a thickener, 1 wt% methanol is added as a heat stabilizer, which can prevent degradation of polymer backbone chain caused by free radicals, and 0.5 mol/L HCl solution is used as a pH control agent to get the pH value to 4–5 and then the Zr crosslinker is added in proportion and stirred for 5–10 min to obtain a polymer fracturing fluid.

### 4.4. Rheological Analysis

Rheological measurements were performed with a Physica MCR302 rheometer (Anton Paar, Graz, Austria). Polymer solution equipped with a coaxial sleeve measuring system. The crosslinked polymer gels were measured using a 25 cm diameter parallel plate system with a 1 mm gap between the two plates.

The apparent shear viscosities of the solutions were obtained by shearing 300 s at a shear rate of 100 s^−1^. The flow curves of the solutions were measured with the shear rate varying from 0.1 to 1000 s^−1^ according to the logarithmic law. Strain sweep tests were performed at a fixed oscillation frequency of 1 Hz with the strain varying from 0.01% to 100%. All tests were performed at 30 °C.

In the measuring sleeve of the rheometer, 20 mL of HPAM base liquid was added. When the temperature reached the required level, pH control agent and heat stabilizer were added and stirred with a glass rod for 1 min. Finally, the crosslinking agent was added and stirred for about 10 s. The rotor was moved to the measuring position and the changes in system viscoelastic modulus during the crosslinking process were observed. The relationship between modulus of polymer crosslinking gel and time at different temperatures (30 °C, 50 °C, 70 °C) and strains (1%, 10%, 30%, 50%) in SAOS mode (f = 1 Hz) were measured. The relationship between modulus of polymer crosslinking gel and time at different temperatures in LAOS mode (f = 1 Hz, γ = 100%) were measured. The temperature increases linearly from 25 °C to 90 °C in 30 min and remains at 90 °C for 30 min.

### 4.5. Thermal Stability Measurements

The temperature- and shear resistance of fracturing fluid gels were evaluated using the HAAKE MARS 60 (Thermo Scientific, Karlsruhe, Germany) rheometer at a constant shear rate of 100 s^−1^. The rheometer was kept sealed during the measurements. In accordance with the “Technical Requirements for Water-based Fracturing Fluids in the Petroleum and Natural Gas Industry (SY/T 7627-2021)”, a high-temperature test program was designed to raise the temperature from 25 °C to the predetermined value within 30 min and then maintain stability for 90 min. If the apparent viscosity of the system is 50 mPa·s during the high-temperature test, the high-temperature resistance of the system is regarded adequate for the specified test temperature; if the apparent viscosity is 50 mPa·s during the test, the high-temperature test can be terminated immediately.

### 4.6. Morphological Analysis

The microscopic morphology of the gels was observed using a QUANTA200 (Thermo Scientific, Karlsruhe, Germany) environmental cryo-scanning electron microscope (cryo-SEM). A small amount of fracturing fluid gel is placed on the sample table, dried under vacuum at −16 degrees and the lens is adjusted to observe image. These images were captured on the surface of the gel at magnifications of 506×.

### 4.7. Molecular Dynamics Simulation

#### 4.7.1. Structural Models

**Figure 19:**
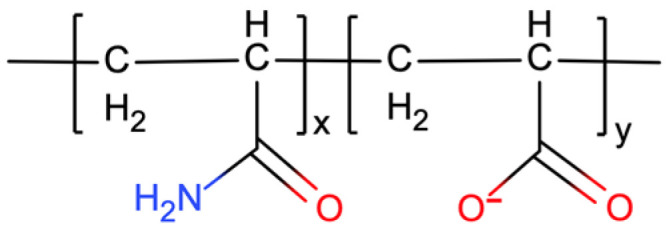
The molecular structure of HPAM in aqueous solutions.

Atactic HPAM chains with polymerization degree 100 were polymerized with acrylamide (AM) and acrylic acid (AA) monomers ($N_{AM}$ = 75 and $N_{AA}$ = 25) according to the structure shown in Figure 19. Packmol software package [48] was used to randomly pack 5 HPAMs into a 10 nm cubic box. The solvent module in GROMACS simulation package was used to fill the cubic box with water solution to a density of 1 g/cm^3^. 125 Na^+^ ions were inserted to neutralize the -COO^−^ negative charge to make the solution net charge zero as a water solution without additional ions. Based on this system, three HPAM solution systems were obtained by inserting different types of salt into the water solution at the same concentration: water solution system (no additional ions), NaCl solution system (0.438 mol/L) and CaCl_2_ solution system (0.438 mol/L).

#### 4.7.2. Simulation Detail

All-atom MD simulations were performed to study the behavior of HPAM in aqueous and salt solutions using GROMACS simulation package (version of 2022.01) [49]. All systems were modeled using Amber ff99SB-ILDN [50] force field together with SPC/E water model. Simulation boxes were set up to place polymer in a water box extending 12 Å. Systems were energy-minimized using steepest descent minimization to remove steric clashes in polymer. Electrostatic interactions were treated with particle mesh Ewald method [51] and van der Waals interactions were calculated using a switching distance of 1.2 nm. Systems were carried out in the NPT ensemble for 20 ns, with the temperature of systems maintained at 300 K using the V-rescale thermostat [52] and the pressure maintained at 1 atm using the Parrinello–Rahman barostat [53] and followed by 50 ns production runs. The integration time step was set to 2 fs. The visualization was prepared using VMD 1.9.2 [54].

## Figures and Tables

**Table 1:** Flow curve fitting parameters for HPAM solutions in various solvent conditions.

C_HPAM_ = 0.6 wt%	Model Parameters				*R*
	$\eta_{0}/\mathrm{mPa}\cdot s$	$\eta_{\infty }/\mathrm{mPa}\cdot s$	λ/s	n	
In DI water	54,310 (±410.75)	7.80 (±0.24)	4.73 (±0.31)	0.07 (±0.01)	0.99
In tap water	36,440 (±270.11)	7.27 (±0.27)	4.29 (±0.12)	0.04 (±0.01)	0.99
In salt solution	586.9 (±13.22)	6.32 (±0.13)	1.82 (±0.03)	0.49 (±0.01)	0.99

**Table 2:** Parameters of HPAM gel crosslinked rheological dynamics model (Equation (4)) at different strains.

γ/%	Model Parameters			
	Tap Water System			
	$G_{max}^{{}^{\prime }}/\mathrm{Pa}$	$G_{min}^{{}^{\prime }}/\mathrm{Pa}$	k/s	R
1	10.38	9.45	0.17	0.98
10	11.19	9.36	0.24	0.98
30	11.54	9.50	0.26	0.99
50	11.62	9.61	0.25	0.99
	salt solution system			
1	1.33	1.05	0.32	0.97
10	1.89	0.87	0.35	0.98
30	3.83	1.13	0.36	0.98
50	3.66	0.73	0.24	0.99

**Table 3:** Parameters of HPAM gel crosslinked rheological dynamics model (Equation (4)) at different temperatures.

T/°C	Model Parameters			
	Tap Water System			
	$G_{max}^{{}^{\prime }}/\mathrm{Pa}$	$G_{min}^{{}^{\prime }}/\mathrm{Pa}$	k/s	R
30	11.54	9.50	0.26	0.99
50	12.95	9.57	0.29	0.97
70	15. 67	9.78	0.30	0.98
	salt solution system			
30	3.83	1.13	0.36	0.98
50	4.52	1.22	0.38	0.98
70	6.41	0.91	0.42	0.99

## Data Availability

Data sharing not applicable.
